# Food Safety Risks Posed by Heavy Metals and Persistent Organic Pollutants (POPs) related to Consumption of Sea Cucumbers

**DOI:** 10.3390/foods11243992

**Published:** 2022-12-09

**Authors:** Edel Oddny Elvevoll, David James, Jogeir Toppe, Esther Garrido Gamarro, Ida-Johanne Jensen

**Affiliations:** 1 Norwegian College of Fishery Science, Faculty of Biosciences, Fisheries and Economics, UiT-The Arctic University of Norway, N-9037 Tromsoe, Norway; 2 Fisheries and Aquaculture Division, Food and Agriculture Organization of the United Nations (FAO), 00153 Rome, Italy; 3 Department of Biotechnology and Food Science, Norwegian University of Science and Technology, NTNU, N-7491 Trondheim, Norway

**Keywords:** food safety, sea cucumbers, heavy metal, contaminants, nutrients

## Abstract

The global production of sea cucumbers was 245 thousand tons in 2020. Sea cucumbers are important food items in Asian and Pacific cuisines, the highest proportion being consumed in China as “bêche-de-mer” dried, gutted, boiled and salted body wall. However, consumption of sea cucumbers is expanding in China and globally, and the high demand has led to decline in populations of sea cucumbers, due to overexploitation. Aquaculture, together with novel fisheries on new species in new regions is easing the demand. Thus, an assessment of food safety is warranted. A literature search on food hazards was performed. A high proportion of the selected papers concerned heavy metals and metalloid hazards, such as mercury (Hg), cadmium (Cd), lead (Pb), and arsenic (As). No specific maximum limits (MLs) have been set for contents of these in sea cucumbers. Thus, the contents were compared with maximum limits set for aquatic animals in general or bivalve molluscs if available. With regard to Hg and Cd levels, none of the samples exceeded limits set by the European Commission or the National Standard of China, while for Pb, samples from highly industrialised areas exceeded the limits. Surprisingly, data on contaminants such as POPs, including dioxins and dl-PCB, PAH and PFAS as well as microbial hazards were scarce. The availability of fresh sea cucumber has increased due to aquaculture. To preserve the original flavour some consumers are reported to prefer to eat raw sea cucumber products, sashimi and sushi, which inevitably causes challenges from the microbial food safety perspective. Altogether, this paper highlights specific needs for knowledge, in particular when harvesting new species of sea cucumbers or in industrialized regions. Systematic monitoring activities, appropriate guidelines and regulations are highly warranted to guide the utilization of sea cucumbers.

## 1. Introduction

A literature search, on hazards challenging the food safety of sea cucumber consumption, was performed in order to assess the status and advise on potential future food safety challenges.

The United Nations has addressed sustainable food security, food safety and enhanced nutrition for decades. Food production is a major greenhouse gas (GHG) emitter. The Intergovernmental Panel on Climate Change (IPCC) paints a rather dark future if we fail to reduce GHG emissions [1]. Warming and acidification of the ocean enhance movement and bio- accumulation, as well as magnification of toxins and contaminants in marine food webs and may reduce marine food productivity and challenge food safety [2]. Shifting human diets from land-based animals (red meat) as a main source of protein to plant based, lower food web sources is a major strategy [3,4]. Seafood is a vital part of a balanced diet and contributes important nutrients such as iodine (I), selenium (Se), vitamins, long chain omega-3 fatty acids and high-quality proteins [5].

The possible contribution of sustainable food (and feed) from well-managed ocean resources, with emphasis put on novel resources from the lower end of the food web, has been put forward many times, most recently by Costello, et al. [6], the Ocean Panel and SAPEA [7]. These recent reports suggest that future gains in world marine culture (and capture) will have to come from expansion and production at lower trophic levels. The role of trophic level in the seafood sustainability discussion, i.e., the relative nutritional benefits, set against the environmental footprint, still needs to be further mapped and ranked against traditional seafood items for more targeted dietary advice [8].

Sea cucumbers are cylindrical echinoderms of the class *Holothuroidea* occurring at various depths. Of more than 1700 species worldwide, around 50–60 are recognised as edible [9,10,11,12]. Tracing the role of sea cucumbers within the global trade is challenging and the number as well as complexity of species from different origins adds to this [13]. Severe underreporting of fisheries exports is also common, as for instance reported exports are less than half of reported imports. This is particularly problematic in developing countries [14]. Rahman et al. 2020 have repeatedly assessed that the world production of sea cucumber increased from 130,000 tons in 1995 to 411,878 tons in 2014 [13] but FAO reports a total capture fishery of *Holothuroidea* increasing from 26 thousand tons live weight in 2007–2016 to 43 thousand tons live weight in 2020 and the aquaculture production of 127 thousand tons in 2010 and to be 202 thousand tons in 2020 [15].

Both markets and consumption of sea cucumbers are centred in East-Asia, with China by far the most important market. The per capita consumption of aquatic products in China has increased from 11.5 kg in 1990 and was projected to reach almost 36 kg in 2020 by FAO [16]. Seafood consumption in Asian countries is driven by a large and growing population, urbanization, increase in income and expansion in seafood production, in particular from aquaculture [16]. In addition, the expansion of international trade of seafood has been growing. China is the ultimate and largest market for sea cucumbers, in addition to traditional consumption by fishers and local markets. In China they are mainly consumed at banquets, as a luxury product a delicacy (i.e. consistency, mouthfeel) gift, or as health supplements [17]. They are found in traditional Chinese medicine pharmacies and are indexed in handbooks of Traditional Chinese Medicine [18]. Sea cucumbers may have huge potential as natural ingredients in foods as supplements and drugs. In addition, beneficial effects, such as wound healing, antitumor, anticoagulant, antimicrobial, and antioxidant activity have been demonstrated. The most studied active compounds being saponins, chondroitin sulfates and glycosaminoglycan but the evidence is growing [19]. However, these have not yet been confirmed by adequate and sufficient animal and clinical trials to allow for nutritional or health associated claims. Thus, such studies are still needed to develop such products from sea cucumbers [19,20]. The expansion of the middle class has led to increased sea cucumber demand and with the association with health, people and families in China now consume sea cucumbers in less formal settings or regularly at home. Thus, domestic production as well as imports have increased tremendously in recent decades [18].

Sea cucumbers are traded fresh, frozen or live. The “bêche-de-mer” (BDM) gutted, boiled and dried body wall, is the most prevalent food product [21]. The production of this traditional dried BDM product utilises low-tech drying processes and enables storing without refrigeration and facilitates production in less developed and remote areas of the world [22]. The dried body wall of sea cucumber is of high nutritional value, rich in protein and in general low in lipids. Sea cucumber has also attracted attention with the increase in health awareness. The content and health effects of a variety of bioactive molecules was reviewed in 2021 by Rasyid et al. [23], by Hossain et al. [24] in 2020 and in 2018 by Pangestuti and Arifin [20,23], and sea cucumbers are suggested to possess anticancer, anti-inflammatory, antimicrobial, antioxidant activities. The documentation of nutritional and health effects from in vitro and in vivo studies was recently reviewed by Liang et al. [19]. They concluded that protective properties against cancer and Alzheimer’s disease have been substantiated, to some extent, by clinical trials, though the numbers of trials were very limited. In addition, they conclude that the promising results from in vivo trials on a range of possible health effects warrant more research into health benefits of extracts and components from sea cucumber [19].

Sea cucumbers have also traditionally been used as medical or functional food; thus, in addition to the food market there has been and still is a growing market for a wide variety of pharmaceutical and nutraceutical extracts [24,25]. 

The high demand in the eastern world has led to decline in populations of sea cucumbers, particularly in Asia and the Indo-Pacific region due to overexploitation [26,27,28,29]. Aquaculture, together with novel fisheries on new species in new regions is easing the demand [28,30]. The Japanese spiky sea cucumber (*Apostichopus japonicus*) has been the most expensive species for a long time [10], and is one of few species that has been cultivated for a while.

Composition varies with species, geography and growth conditions as well as postharvest processing. Sea cucumbers are known as deposit or suspension feeders, feeding on suspensions near the bottom, sediments and deposited organic matter. Thus, sea cucumbers absorb nutrients and contaminants from their ocean habitat and play a key role in nutrient recycling [31].

Sediments are associated with higher concentrations of contaminants and heavy metals. The levels in marine sediments are a consequence of in situ geochemical processes, draining from flooded soils, sewage and agricultural, tourist and industrial activities in and around the sites/locations of both harvesting and farming of sea cucumbers. Combined with a suggested lower metabolising capacity or bio-accumulation of POPs [32] and heavy metals [33,34], consumption of sea cucumber may have negative impacts for human consumers.

Since neither the European Commission, nor the Codex Alimentarius [35] or the National Standard of China have set *specific* maximum limits (MLs) for sea cucumber on contents of heavy metals (or persistent organic pollutants POPs), the hazard assessment was conducted by comparing contents of heavy metals with MLs given eighter by the European Commission [36] or the MLs given for contents in “bivalves” by the National Standard of China (NSC) [37] for contents in “Aquatic animals and its products”. The latter MLs used as China is the prime market of sea cucumber. A similar strategy to assess food safety of sea cucumber consumption was used by Montero et al. (2021), Sicuro et al. (2012) and Storelli et al. (2001) [26,33,38]. In addition, when both global (Codex Alimentarius) and regional (EC) MLs are missing, as for inorganic (iAs), the content of iAs in the sea cucumber are compared with the non-specific ML (Aquatic animals and products) given by NSC and the consumption rate limits (CR_lim_) are calculated from the he lower end benchmark dose (BMDL) of suggested by European Food Safety Authority (EFSA) expert panel [39].

The present review aims to briefly summarize data on sea cucumbers nutritional composition, and in particular collect and review data related to food safety. Available literature on contents of dioxins, polychlorinated biphenyls (PCB), dl-PCBs, poly aromatic hydrocarbons (PAH), per- and polyfluoroalkyl substances (PFAS and PFOS) and heavy metals, as well as microbial and physical hazards, that may pose food safety risks at consumption of sea cucumber, have been collected and possible health impacts of the hazards are assessed.

## 2. Data Sources and Search Strategies

**Figure 1:**
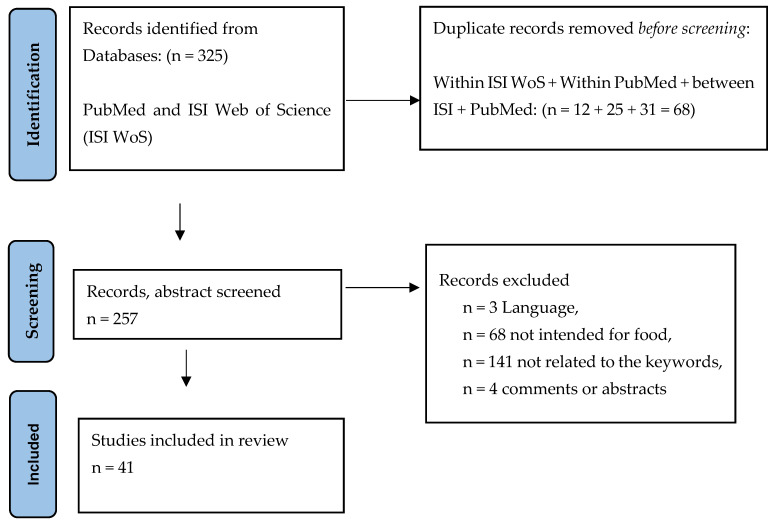
Flow chart of the identification of studies via databases (ISI Web of Science and PubMed), the selection of studies of food safety risks posed by heavy metals and persistent organic pollutants (POPs), by consumption of sea cucumbers. Adjusted PRISMA for the current study, more information https://www.bmj.com/content/372/bmj.n71 (accessed 14th November 2022).

A systematic search of the literature, performed on 7 March 2022, via two databases—papers indexed in Web of Science and PubMed—were screened from 1900, to the present (Figure 1). No language limitation was added. The following keywords were used; “food safety” or “hazard” or «contaminant» or “dioxin” or “PCB” or “PAH” or “heavy metal” each combined with «sea cucumber». The literature searches generated 325 hits. The papers were identified, duplicates within (12 ISI, 25 PubMed) and between the two databases (31) were removed (n = 68), and the abstracts of the residual papers (n = 257) were then screened to confirm relevance.

In addition, data on import notification were checked in the FAO database, FishStatJ [40]. The FAO dataset contains rejections, detentions, recalls, and issues reported by competent authorities in Australia, the European Union, Japan, and the United States of America. The data shown in this dataset is extracted from the following publicly available import notification portals: the Imported Food Inspection Scheme of the Australian Government, the Rapid Alert System of Food and Feed Portal in the European Union, the website of the Ministry of Health Labour and Welfare of Japan and the Import Refusal Report of the Food and Drug Administration of the United States of America. No food safety issues were reported in these portals for sea cucumber or products containing sea cucumber over the past 7 years.

### Eligibility Criteria

The following eligibility criteria were used to select studies or papers: (i) published as a full original article with full text; (ii) studies of species of sea cucumber intended for food. Studies were excluded if: (i) the article was not in English (n = 3); (ii) sea cucumber was not intended as a food product (n = 68); (iii) species of sea cucumber not specified (n = 0); (iv) the paper was not related to the keywords (n = 141) or (v) review articles, comments or abstracts only (n = 4). All together two hundred and fifty-seven abstracts were screened for eligibility by two authors (E.O.E/I-J.J.) and 41 articles were included. Of the forty-one papers selected for further study, twenty-three were on heavy and trace metals, ten concerned persistent organic pollutants (POPs), dioxins and PCBs, PFAS, including PAHs and Lindane, two on microbial load related to food safety, three on antimicrobial contamination and two on contaminants from household and personal care products. Only one paper on contents of physical hazards, metal and glass, as well as plastics (incl. micro—and nano plastics), related to food safety or contaminants were retrieved by these searches.

A high proportion (23 out of a total of 41) of the retrieved studies, by abstract, were related to the content of heavy and trace metals in sea cucumber. Thirteen of these contained data on the content of heavy metals in food products and thus were of particular relevance to this work (Figure 1). In addition, two papers explored the speciation of As and Hg in sea cucumber and are thus of particular relevance [41,42] for the assessment of toxicity. Surprisingly enough, since sea cucumbers are filter feeders, no papers regarding toxin or biotoxins in any combination with the term “food safety” were retrieved in the literature search. All hits linked to toxin or biotoxin were related to the survival or to disease in the organisms, the sea cucumbers.

## 3. Results

### 3.1. Proximate Composition

The proximate composition (water, ash, protein and fat) of sea cucumber varies with species (Table 1). In a study of the nutritional composition of eight common processed sea cucumber species the protein contents ranged from 40.7 to 63.3%, the fat content from 0.3% to 10.1%, and ash content varied between 15.4% and 39.6% [43]. These results are similar to what was found in a study of nutritional value of processed *Holothuria scabra,* 41.0–60.2% protein, 1.7–2.3% fat, and 29.8–40.0% ash [44]. The nutritional content of sea cucumber varies with species, season and habitat as well as age. A recent paper, from 2019, on nutrients in fresh sea cucumber (*Holoturia tubulosa*) body wall by Künili and Çolakoğlu [45], showed seasonal variations in water (81–87%), protein (8–10%), lipid (1.6–1.9%) and ash (4.1–5.1%). The water or moisture content was similar or slightly higher than those reported for other sea cucumbers. The protein content was similar to other studies but the lipid content was higher [23,46].

### 3.2. Lipids

Seafood consumption has been documented as reducing the incidence of cardiometabolic diseases [47,48], primarily through epidemiological evidence and meta-analyses [49] but also by preclinical and clinical studies on the impact of long-chain marine omega-3 polyunsaturated fatty acids (PUFAs), EPA (eicosapentaenoic acid; 20:5 n−3) and DHA (docosahexaenoic acid; 22:6 n−3) and lipid-soluble components present in seafood [50,51]. Recommendations to consume seafood are often based on the content of EPA and DHA and the World Health Organization (WHO) recommends sufficient seafood consumption to provide an average intake of 200–500 mg of EPA and DHA per day [52]. Sea cucumbers are in general low in lipids and marine PUFA’s compared to other seafood items [23]. In general sea cucumbers are lean with lipid content of less than 2% for most species, but can vary from 0.1–3.5% in fresh weight and up to 10% in dry weight. Arachidonic acid (AA) and EPA have been reported as major fatty acids in several species, ranging from 7–20% and 4–16%, respectively [23]. In a review by Wen et al., they showed that the fatty acid profile varied between species, diet and ambient temperature, as well as being affected by the drying procedure (gutting, boiling and drying) [43].

### 3.3. Proteins

Proteins are essential nutrients for growth and development. Both the quantity and quality of protein are important when evaluating a protein source. The protein content of fresh sea cucumbers is quite low and vary between 3.4–8.7%, whereas dried sea cucumbers contain between 37–72% protein. The quality of protein resources is usually described by different metrics to take into account factors such as digestion, absorption and assimilation capacity. One method of assessing the quality of proteins is by chemical score. Chemical score is the lowest ratio between each EAA in the food protein and the corresponding EAA in a reference protein, as proposed by FAO/WHO. Proteins of animal source normally have a chemical score of 1.0, while cereal proteins normally range from 0.4 to 0.6. Legumes, beans and nuts normally fall between these. In general seafoods have high protein quality with contents of all essential AA above the reference-scoring pattern for adults [53], which indicates that the protein quality is superior to most terrestrial plants and comparable to terrestrial animal protein. Although the protein content of fresh sea cucumbers is low, the protein quality is high, and all EAA are found in sea cucumbers. *Holothuria tubulosa* [45] was high in glycine and alanine followed by threonine, glutamic acid, aspartic acid and arginine and this is consistent with other studies on the same species [38]. Glycine has been considered the dominant amino acid in many sea cucumber species. Variations in amino acids, probably due to species, feed, life stage and environment are reported [23,46,54,55].

The ratio of EAA to nonessential amino (NEAA) is another indicator of protein quality and the EAA:NEAA ratio found by Kunili et al. [45], 0.40–0.45, which is close to what may be regarded as optimal, was also reported by Sicuro et al. [38] for *Holothuria tubulosa*. This ratio varies, between 0.25 and 0.60 [55] as has been reported for other sea cucumbers. The chemical scores were higher than reference amino acids, except for lysine [45]. The total sulfur amino acid (methionine + cysteine) was close to the recommended level of 20 mg/kg (per kg of body weight) for adults by FAO (2007). The total aromatic amino acids were also similar to recommended values [45].

The high level of small amino acids such as glycine and alanine is likely to reflect a high level of collagen in the body wall, giving a particular texture and mouthfeel [56]. In the study carried out by Rasyid et al., [23] glycine and glutamic acid were reported to be the main free amino acids in sea cucumbers in addition to aspartic acid and alanine, all known to enhance flavour in foods [57].

### 3.4. Minerals

Some minerals and trace elements, essential in tiny amounts, are more abundant in seafood than in terrestrial animals. Particularly Se and I, but also zinc, magnesium and calcium are high in selected seafoods [58,59,60].

Sea cucumbers are echinoderms and possess an endoskeleton, rich in minerals, in particular calcium, as a part of the edible portion. Information about mineral content in sea cucumbers is limited. Analyses of calcium, potassium, and magnesium have suggested sea cucumbers are a natural source of minerals. The mineral content varies by species, season, maturity, age and feed. In addition, sea cucumbers are reported to contain vitamins, including A, B1, B2, and B3 [25].

#### 3.4.1. Selenium

Individuals in many European countries do not reach the recommended dietary intake of Se of 70 μg/day [61]. Low dietary intake of Se has been associated with poor immune function, cognitive decline and increased risk of autoimmune thyroiditis, Graves’ disease, goitre, and mortality. Selenium intake has also shown antiviral effects, protection from infection and is essential for successful male and female reproduction [62]. Selenium is known for the antioxidant properties of selenoenzymes, and is essential for the activation of the thyroid hormone [62]. Selenium has also been suggested as protecting against Hg toxicity by decreasing the bioavailability of Hg. Experimental studies have indicated that a Se:Hg molar ratio ≥1 in seafood may offer protection against Hg toxicity [63]. It is also noteworthy that although Se is an essential mineral, adverse health effects have also been observed at very high intakes.

The concentration of Se was measured in two of the thirteen studies listed in Table 2. Sicuro et al. [38] analysed Se in the edible tissues of *Holothuria polii* (4.24 ± 0.04 mg/kg dw) and *Holoturia tubulosa* (4.18 ± 0.16 mg/kg dw) collected in the Mediterranean Sea. Wen and Hu [55] analysed eleven products of species (*Stichopus herrmanni*, *Stichopus chloronotus*, *Thelenota ananas*, *Thelenota anax*, *Holothuria scabra*, *Holothuria mexicana*, *Holothuria fuscogilva*, *Holothuria fuscopunctata*, *Actinopyga mauritiana*, *Actinopyga caerulea* and *Bohadschia argus*), purchased at a local retail market and supermarket in Guangzhou, China, where the range of Se was 0.9–3.7 mg/kg dw and in general lower than the Mediterranean samples. This may be attributed to species, geography and probably the difference in post-harvest handling and processing. Sea cucumbers are excellent sources of Se when compared to other seafood items [64].

#### 3.4.2. Iodine

Iodine (I) is a vital regulator of metabolic rate as well as physical and mental development [65]. Iodine deficiency is still a public health concern even if salt iodization has had an impact. Europe is also the continent with the highest prevalence of I deficiency and 44% of schoolchildren have inadequate I status according to the World Health Organization [65]. Mild I deficiency during pregnancy can have long-term adverse impacts and emphasis should be placed on adequate I intake prior to pregnancy, during pregnancy and when lactating [66]. However, excess iodine intake can cause dysfunction of the thyroid gland. EFSA’s Scientific Committee on Food (SCF) suggested a tolerable upper intake level (UL) for adults of 600 μg I/day and adjusted this for the remaining age groups, based on differences in body weight [67]. The information on I in sea cucumbers is largely absent but a recent paper on the design of functional food products meeting nutritional needs for older people utilises extracts from holothurians (*Parastichopus tremulus* and *Holothuria forskali*) as a source of minerals (calcium, phosphorus and magnesium) in general, and easily available I and Se in particular [68].

#### 3.4.3. Heavy Metals and Trace Elements

The micro-minerals can be divided into toxic, hazardous and regulated compounds, and those not toxic and thus not regulated. A number of the minerals seem not to have guidelines for maximum levels or regulation and the significance of such data is unclear. Wen and Hu [55] assessed the nutritional quality and the potential hazards of sea cucumber products, by calculating the content per 100 g serving and compared with the recommended intake and limits set by international authorities. Average levels of the essential elements Na, Cl, K, Ca, Mn and Cr were compared with the daily adequate intakes (AI), Mg, Fe, Cu, Zn and Se with the daily dietary reference intake (DRI), and Na, Cl, Ca, Mn, Fe, Cu, Zn and Se were compared with the daily tolerable upper intake levels (UL). The AI, DRI and UL used were set by the US Department of Agriculture (USDA) for adult females and males. Arsenic was evaluated in terms of the action level (AL; similar in meaning to the ML) set by the US Food and Drug Administration (USFDA). When the daily intake recommendations and levels in the products (body wall) from eleven different sea cucumbers were assessed they [55] found sea cucumbers to be good sources of Na, Cl, Mg, Ca, Fe, Cu, Se, and Cr.

The level of contaminants or heavy metal levels (Cd, Hg, As and Pb) in the selected papers were compared to the maximum allowed levels (ML) for contaminants given in food standards such as (China National Food Safety Standard, GB 2762-2017), the maximum levels for certain contaminants in foodstuffs European Commission regulation (EC) No 1881/2006 [36], or the Codex general standard for contaminants and toxins in food and feed (Codex Standard 193–1995, often abbreviated to the CAC) [35]. These regulatory levels are given in wet weight and thus lack of experimental data on wet weight, or access to moisture contents challenges exposure assessments for such hazards.

## 4. Hazards

### 4.1. Food Safety and Risk Assessments

To assess food or product safety of sea cucumbers the MLs for other seafood items [36,69,70,71] may be used. Lack of data on daily intake of sea cucumber (quantities, frequencies, product used) add to the challenge of assessing risks of sea cucumber consumption. Human health risk assessment is thus missing and data gaps such as more rigid analysis of contaminants, and an overview of the consumption needs to be done to enable a valid health risk assessment of sea cucumber consumption. Thus, calculating the maximum tolerable daily or weekly sea cucumber consumption/intake based on available limits of oral exposure without enhanced lifetime risk of diseases (TWI, PTWI, RfD or BMDL) may be used as an approach to guide consumers. In addition, body weight of individuals—children, youths and adults—as well as age (fertility, pregnancy) should be taken into account to ensure that vulnerable groups in the population are protected.

The most prevalent sea cucumber food product is “bêche-de-mer” body wall, gutted, boiled and dried. The concentration of metals in dried products is about 5–10 times higher than in fresh or rehydrated. The lack of rigid experimental data on wet weight, or access to moisture contents in the samples or consumed products, challenges exposure assessments for such hazards.

A high proportion of the retrieved studies, by abstract, were related to the content of heavy and trace metals (n = 23) in sea cucumber. Thirteen of these contained original experimental data on content of heavy metals in food products and thus were of particular relevance to this work (Table 2). In addition, two papers explored the speciation of As (Table 3) and Hg in sea cucumber and are thus of significant importance [41,42] for the assessment of toxicity.

### 4.2. Chemical Hazards, Heavy Metals

The ability of sea cucumber to absorb and accumulate minerals and trace elements from the local environment results in sea cucumber potentially being an important source of macronutrients but also entail risks of being exposed to harmful elements when consumed. Specific heavy metals, such as Hg, Cd, As and Pb, are known to present food safety concerns in other seafood products [72,73], and are thus regulated. No specific maximum limits (ML) have been set, by the European Commission (EC) or the Codex (CAC) for contents of Hg, Cd, As and Pb for Echinodermata [35,36]. Thus, the levels present in sea cucumbers were compared with limits for food items from aquatic animals, and if possible, other sedentary filter feeders such as bivalve mollusks from CAC and EC.

**Table 2:** Species, number of samples n, geographical region of sampling, concentration of Mercury (Hg), Cadmium (Cd), Arsenic (As) and Lead (Pb) given as mean ± standard deviation (SD) mg/kg dry weight (dw) in body wall or comparable tissue of sea cucumber.

Species		Geographical Region	Mercury	Cadmium	Arsenic	Lead	Paper
*Acaudina leucoprocta*	n = 3	East China Sea	0.06 ± 0.01	0.05 ± 0.01	5.64 ± 0.24	1.38 ± 0.21	Lin et al. [38]
*Actinopyga caerulea*	n = 3	Guangzhou, China. Market samples	nd	0.06 ± 0.01	3.3 ± 0.1	0.15 ± 0.01	Wen & Hu [55]
*Actinopyga mauritiana*	n = 3	Guangzhou, China. Market samples	nd	0.05 ± 0.01	2.1 ± 0.1	0.11 ± 0.01	Wen & Hu [55]
*Apostichopus japonicus*	n = 3	Bohai Sea Yellow Sea. Range eight farming sites	na	0.31–0.85	4.26–12.39	1.05–4.25	Mohsen et al. [74]
	n = 3	Panshan, Bohai Sea Yellow sea	na	0.85 ± 0.02	10.47 ± 0.28	2.18 ± 0.36	Mohsen et al. [74]
	n = 3	Lvshunkou, Bohai Sea Yellow Sea	na	0.38 ± 0.03	10.88 ± 0.29	2.59 ± 0.21	Mohsen et al. [74]
	n = 3	Rongcheng, Weihai, Bohai Sea Yellow Sea	na	0.36 ± 0.04	5.99 ± 0.11	1.05 ± 0.13	Mohsen et al. [74]
	n = 3	Haiyang, Yantai, Bohai Sea Yellow Sea	na	0.82 ± 0.03	5.38 ± 0.83	1.76 ± 0.07	Mohsen et al. [74]
	n = 3	Chengyang, Qindao, Bohai Sea Yellow Sea	na	0.57 ± 0.05	5.25 ± 0.42	2.72 ± 0.08	Mohsen et al. [74]
	n = 3	Pingdao island, Rizaho, Bohai Sea Yellow Sea	na	0.31 ± 0.03	12.39 ± 0.25	1.56 ± 0.25	Mohsen et al. [74]
	n = 3	Tangshan, Bohai Sea Yellow Sea	na	0.54 ± 0.02	9.86 ± 0.11	1.94 ± 0.15	Mohsen et al. [74]
	n = 3	Laizhou, Bohai Sea Yellow Sea	na	0.50 ± 0.01	4.26 ± 0.87	4.25 ± 0.23	Mohsen et al. [74]
	n = 3	North Yantai region of China	na	0.32 ± 0.07	na	na	Liu et al. [75]
	n = 63	South Korea	0.002 ± 0.002	0.05 ± 0.07	2.24 ± 1.82	0.06 ± 0.05	Choi et al. [76]
*Bohadschia argus*	n = 3	Guangzhou. China. Market samples	nd	0.03 ± 0.01	3.2 ± 0.1	0.13 ± 0.01	Wen & Hu [55]
*Cucumaria frondosa*	n = 3	Guangzhou Qingping, China, Canadian S2	0.17 ± 0.01	0.61 ± 0.05	3.59 ± 0.39	0.57 ± 0.04	Song et al. [77]
	n = 3	Guangzhou Qingping, China, Canadian S4	0.15 ± 0.00	0.40 ± 0.37	3.13 ± 1.12	0.08 ± 0.02	Song et al. [77]
	n = 3	Guangzhou Qingping, China, Canadian S6	0.17 ± 0.00	0.43 ± 0.03	2.58 ± 0.14	0.53 ± 0.04	Song et al. [77]
	n = 3	Guangzhou Qingping, China, Canadian S7	0.17 ± 0.01	0.32 ± 0.09	2.52 ± 0.17	0.45 ± 0.04	Song et al. [77]
*Eupentacta fraudatrix*	n = 6	Peter the Great Bay, Sea of Japan 2016	na	0.15 ± 0.01	na	17.08 ± 1.12	Dolmatova et al. [78]
	n = 5	Peter the Great Bay, Sea of Japan 2008	na	0.38 ± 0.03	na	5.01 ± 0.66	Dolmatova et al. [78]
	n = 5	Peter the Great Bay, Sea of Japan 2008	na	0.24 ± 0.01	na	nd	Dolmatova et al. [78]
	n = 6	Peter the Great Bay, Sea of Japan 2016	na	0.36 ± 0.04	na	nd	Dolmatova et al. [78]
*Holothuria floridana*	n = 8	Cispatá Bay, Colombia. Range seven sites	0.074–0.090	0.016–0.026	na	0.030–0.038	Marrugo-Negrete et al. [79]
*Holothuria fuscogilva*	n = 3	Guangzhou, China. Market samples	nd	0.03 ± 0.01	3.8 ± 0.1	0.13 ± 0.01	Wen & Hu [55]
*Holothuria fuscopunctata*	n = 3	Guangzhou, China. Market samples	nd	0.03 ± 0.01	6.1 ± 0.1	0.56 ± 0.03	Wen & Hu [55]
*Holothuria leucospilota*	n = 30	Qeshm Island, Persian Gulf	na	0.29 ± 0.14	na	21.39 ± 2.52	Mohammadizadeh et al. [80]
	Range	Qeshm Island, Persian Gulf. Range three sites	na	0.16–0.45	na	19.09–23.24	Mohammadizadeh et al. [80]
*Holothuria mexicana*	n = 3	Guangzhou, China. Market samples	nd	0.04 ± 0.01	2.0 ± 0.1	0.69 ± 0.06	Wen & Hu [55]
*Holothuria polii*	n = 19	Gulf of Cagliari, Foxi, Sardinia, Italy	0.023	0.03	22.9	0.88	Montero et al. [30]
	n = 5	Southern Adriatic Sea, Italy, Pooled samples of 2 kg	na	0.07 ± 0.0	33.30 ± 0.85	0.65 ± 0.03	Sicuro et al. [42]
	n = 500	Southern Adriatic Sea, Italy	0.96 ± 0.022	0.04 ± 0.01	na	1.26 ± 0.12	Storelli et al. [37]
*Holothuria scabra*	n = 3	Qeshm Island, Persian Gulf	na	0.15 ± 0.02	na	1.92 ± 0.49	Mohammadizadeh et al. [80]
	Range	Qeshm Island, Persian Gulf. Range three sites	na	0.13–0.17	na	1.52–2.55	Mohammadizadeh et al. [80]
	n = 3	Guangzhou, China. Market samples	nd	0.04 ± 0.01	1.1 ± 0.1	0.56 ± 0.03	Wen & Hu [55]
*Holoturia tubulosa*	n = 15	Gulf of Cagliari, Giorgino, Sardinia, Italy	0.043	0.02	18	0.44	Montero et al. [30]
	Range	Dardanelles Strait, Turkey. Range three sites	na	0.04–1.66	na	0.48–5.80	Turk Culha et al. [81]
	n = 5	Southern Adriatic Sea, Italy. Pooled samples of 2 kg	na	0.07 ± 0.0	22.35 ± 0.08	1.16 ± 0.04	Sicuro et al. [42]
*Stichopus chloronotus*	n = 3	Guangzhou, China. Market samples	nd	0.06 ± 0.01	1.7 ± 0.1	0.12 ± 0.01	Wen & Hu [55]
*Stichopus herrmanni*	n = 3	Guangzhou, China. Market samples	nd	0.03 ± 0.01	2.5 ± 0.1	0.52 ± 0.03	Wen & Hu [55]
*Thelenota ananas*	n = 3	Guangzhou, China. Market samples	nd	0.03 ± 0.01	5.1 ± 0.1	0.55 ± 0.03	Wen & Hu [55]
*Thelenota anax*	n = 3	Guangzhou, China. Market samples	nd	0.04 ± 0.01	2.8 ± 0.1	0.25 ± 0.02	Wen & Hu [55]

Below the detection limit (BLD) = nd; Not analysed = na.

**Table 3:** Species, body part selected and mean total arsenic (As) and recovered inorganic arsenic (iAs) concentration in mg/kg dry weight (dw) ± standard deviation (SD) in body wall of sea cucumbers *Cucumaria frondosa* (n = 3)*, Apostichopus californicus* (n = 5) *and Apostichopus japonicus* (n−1). Adapted from Gajdosecheva et al. Tables 1 and 2 in [42].

			Recovered	Recovered
Species	Body Part and Location	Total As	iAs	Total As
*Cucumaria frondosa*	Body wall NS	6.0 ± 1.1	1.4 ± 0.17	2.0 ± 0.27
	Body wall NL	5.2 ± 0.8	1.7 ± 0.41	2.3 ± 0.34
	Process Body wall NS	4.3 ± 1.6	0.63 ± 0.24	0.93 ± 0.17
*Apostichopus californicus*	Muscle BC	36.0 ± 3.5	0.017 ± 0.012	4.3 ± 1.1
	Skin BC	28.0 ± 0.5	0.042 ± 0.054	0.85 ± 0.20
*Apostichopus japonicus*	Body wall Liaoning	5.0 ± 0.1	0.069	1
	Body wall Shandong	7.1 ± 0.04	0.13	1.9
	Body wall Fujian	7.7 ± 0.09	0.12	1.1

NS Atlantic coast, Nova Scotia, Canada; NL Newfoundland and Labrador, Canada; BC Pacific coast, British Colombia, Canada; Yellow Sea, East China, Liaoning, Shandong and Fujian Provinces.

#### 4.2.1. Mercury

Mercury (Hg) and methylmercury (MeHg) are potent heavy metal toxins able to cross the placenta as well as the blood-brain and the blood-cerebrospinal fluid barriers [73]. MeHg and ethylmercury (EtHg) have stronger cytotoxic effects than inorganic Hg, as they are lipid soluble and more easily absorbed. Critical targets for toxicity include the kidney, liver, nervous system, immune system, reproductive and developmental systems. The Hg content varies widely and is in general higher in predatory fish, ie those higher in the food chain. More than 90% of the total Hg in seafood is MeHg and seafood is the primary source of MeHg exposure for humans of all ages in Europe. A tolerable weekly intake (TWI) of 1.6 μg/kg bw/w for MeHg (CAC) and 1.3 μg/kg bw/w for MeHg (EFSA) was set based on prenatal neurodevelopmental toxicity [35,74]. The maximum content of Hg, in unspecified fish muscles and fishery products, including molluscs and crustaceans is set at 0.5 mg/kg wet weight (ww) in fish, crustaceans and molluscs. Higher limits exist, for instance for tuna (1.2 mg/kg), alfonsino (1.5 mg/kg), marlin (1.7 mg/kg) and shark (1.6 mg/kg), by the Regulation of Codex Alimentarius 1995, or halibut (1.0 mg/kg ww), a long lived bottom fish, by (EC) No 1881/2006 [36], amended by the Regulation (EC) No 629/2008.

Six of the included studies (Table 2) included Hg in their analysis, Wen and Hu [55] were not able to detect Hg, below the detection limit (BLD). The content varies and this may be attributed to species, location, as well as degree of post-harvest handling and processing. The range in concentration varied from 0.002 mg/kg dry weight (dw) in *Apostichopus japonicus* from South Korea [75] to 0.96 mg/kg dw in *Holothuria polii* from the Southern Adriatic Sea, Italy [33] though none of them exceeded the limits set by the European Commission [36], 0.5 mg/kg ww in unspecified fish muscles and fishery products, including molluscs and crustaceans. Liu et al., 2016 found organic MeHg in all samples of *Apostichopus japonicus,* none of the samples exceeded the limits set by the European Commission or National Standard of China GB 2762–2012 being below the maximum value allowed in non-predatory fish species of 0.5 mg/kg Met-Hg [41].

#### 4.2.2. Cadmium

Cadmium exposure has been reported to be associated with delayed growth in early childhood and with adverse effects on neurodevelopment and cognitive function in children [76,77]. In 2011 JECFA set a provisional tolerable monthly intake (PTMI) for Cd of 25 µg/kg body weight per month, which corresponds to a weekly intake of 5.8 μg/kg bw and reflects the long half-life of Cd in humans [78]. For Cd a maximum limit of TWI 2.5 μg/kg bw/w has been established by EFSA. The European maximum levels of Cd is set at 0.05 mg/kg ww in the muscle of most/unspecified fish, but as high as 1.0 mg/kg ww for bivalve molluscs [36].

All the thirteen studies (Table 2) included Cd in their analyses, with levels ranging from 0.03 mg/kg dw in *Bohadschia argus* [55] to 0.9 mg/kg dw in *Apostichopus japonicus* [79], both the highest and the lowest levels were found in samples from Chinaa. No systematic variation depending on area was found, but the content was as expected influenced by industrial activity [38]. The concentration of metals in dried products are 5–10 times higher than in fresh or rehydrated [26]. The lack of rigid experimental data on wet weight, or alternatively access to water or moisture contents in the samples of consumed products of *Cucumaria frondosa* challenges assessments of the concentration versus the limits. But none of the samples exceeded the limits set, 1.0 mg/kg ww, for bivalve molluscs [36].

#### 4.2.3. Arsenic

There are over 100 As species naturally occurring in the environment and As toxicity varies among its species with iAs as a Class 1 carcinogen and organic forms like Arsenobetaine exhibiting low toxicity, as reported by the working group of the International Agency for Research on Cancer (IARC, 2012) [80]. Arsenic is highly toxic in its inorganic form and is naturally present at high levels in groundwater in some countries/regions. Arsenic contaminated water may lead to long-term exposure associated with skin lesions, cancer, developmental toxicity, neurotoxicity, cardiovascular diseases, abnormal glucose metabolism, and diabetes [39,78].

There is no EC ML for As in seafood intended for human consumption. The ML of iAs in food is only specified for rice and rice products [81]. In 2009, EFSA requested a revision of the then Provisional Tolerable Weekly Intake (PTWI) of iAs from all sources (0.015 mg/kg body weight) as it was no longer considered health protective and they established a range from the benchmark dose (BMD) lower confidence limit (BMDL). At 1%, BMDL_01_ values between 0.3 and 8 μg/kg b.w. per day an association was identified with cancers of the lung, skin and bladder, as well as skin lesions. Maximum daily dietary iAs intake should be below 0.3 μg/kg b.w.; for a 60 kg body weight adult, the recommendation would then be a maximum of 18 μg iAS/day [39]. Based on the limited data on iAs in published literature, EFSA used fixed values for iAs of 0.1 mg/kg ww in seafood (shellfish, molluscs etc.) when calculating human dietary exposure. Later in 2010, JECFA performed a risk assessment based on epidemiological studies of lung cancer rates and proposed a BMDL_05_ value of 3.0 μg/kg b.w. (2–7 μg/kg bw/day based on the range of estimated total dietary exposure) using a range of assumptions to estimate total dietary exposure to iAs from drinking-water and food [78,82]. Organic As is generally assumed to be of no toxicological concern or far less toxic than iAs species [78,82,83]. Arsenic in foods of marine origin is mainly present in the form of organic compounds which are less acutely toxic than the toxic inorganic ones.

The abundance of total As species in seafood in general, and also in sea cucumbers, is highly variable (Table 2). Seven of the thirteen studies included total As in their analysis with levels ranging from 1.1 ± 0.1 mg/kg dw in Chinese market samples of body wall from *Holothuria scabra* [55] to 33.3 ± 0.9 mg/kg dw in similar samples of *Holothuria polii* [26] harvested in the Southern Adriatic Sea, Italy.

There are no guideline values, maximum levels (ML), for total As in seafood in Europe, but total As concentrations found in tissues of *Cucumaria frondosa* (3.6 ± 0.4 mg/kg dw) in Chinese market samples [84] or (6.0 ± 1.1 mg/kg dw), (Table 3) in samples harvested on the Atlantic Coast, Nova Scotia, Canada are lower than those reported by Montero et al., 2021 [26] in *Holothuria tubulosa* (18.0 mg/kg dw or 2.2 ± 0.5 mg/kg ww) and *Holothuria polii* (22.9 mg/kg dw or 3.7 mg/kg ww) from the Southern Adriatic Sea, Italy. To make a full assessment we would need additional data on speciation.

Levels of total As ww in the sea cucumber as calculated by Montero et al., 2021 [26] are similar to commonly consumed fresh seafood (molluscs 4.0 ± 3.6 mg/kg ww; pelagic fish 6.5 ± 7.2 mg/kg ww; demersal fish 5.1 ± 5.4 mg/kg ww) [85], sampled along the Mediterranean coast and are thus assessed as suitable for human consumption.

As mentioned, As in seafood is in generally present in the form of organic compounds but some organisms may contain high levels of iAs. Speciation for each product is thus warranted and indeed some of the sea cucumbers are exceptions to this. A recent paper by Gajdosecheva et al. 2020 [42] was dedicated to the analyses of total As and the specimens of As in different organs (body wall, tentacles, internal organ, skin and muscle) of sea cucumber. To enable the extraction of inorganic and water soluble As species sequential extraction was used and the extraction efficiency or recovery was validated by the use of a cuttlefish certified refence material (SQUID-1) as an internal standard [42]. Both total As and As species concentration varied significantly between sea cucumbers species, as well as between the sampled individuals within the species (Table 3). 

Total As, in the edible part, the body wall of *Cucumaria frondosa* ranged between 4.3 ± 1.6 and 6.0 ± 1.1 mg/kg dw. The lowest being the processed body wall or the product to be consumed. The total As level in muscle tissue of *Apostichopus californicus* harvested from the Pacific coast was measured to 36 ± 3.5 mg/kg dw and was comparable to *Holothuria polii* with a level of 33.3 ± 0.9 mg/kg dw, highest of the studies referred in Table 2 [26]. The organic form, was the most abundant As species in *Apostichopus californicus* and *Apostichopus japonicus*. On the contrary, inorganic iAs represented around 70% of the total *recovered* As in the body parts of *Cucumaria frondosa* harvested in Nova Scotia (NS) or Newfoundland and Labrador (NL).

Using a maximum daily dietary iAs intake of 0.3 μg/kg body weight; for a body weight of a 60 kg adult, a maximum of 18 μg iAs/day would limit the intake of the most prevalent food product of sea cucumber “bêche-de-mer” or processed body wall of *Cucumaria frondosa* to 29 g, and body wall to 11 (NL) g and 13 (NS) g [42]. All consumption patterns assessed as highly unlikely. These iAs levels are given on a dry weight basis, or 5–10 times higher than the rehydrated consumed items, and the low recovery (around 30%) in the speciation analysis, challenges an assessment of maximum intake of the products as well as possible exposure assessments.

Worldwide regulations for As and iAs in food were recently reviewed in detail by Petursdottir et al., 2015 [86] and for the most prevalent market, that of China, a maximum level of iAs 0.5 mg/kg wet weight on “Aquatic animals and products (excluding fish and fish products)” has been set in 2012 [37].

Altogether, there are relatively few studies on As in sea cucumbers and very limited knowledge on As speciation. The high proportion (70%) of iAs of total As in *Cucumaria frondosa,* even if the low recovery and large standard deviation between samples are taken into account, highlights a need for As speciation data as well as monitoring activities. Sea cucumbers are harvested globally and data are not available to provide a global overview or a thorough risk assessment of consumption.

#### 4.2.4. Lead

Lead is a cumulative toxicant, particularly harmful to young children. It affects multiple body systems, the brain, liver, kidney and bones. Lead may accumulate in teeth and bones over time. It is released by blood during pregnancy and transferred via the placenta to the fetus. In 2013, the CONTAM Panel concluded that the current PTWI of 25 μg/kg b.w. is no longer appropriate, as there is no evidence for a threshold for critical Pb-induced effects and that the exposure, from all sources, should be kept as low as possible [87]. The European maximum limit of Pb is set at 0.3 mg/kg ww in the muscle of most fish species, but as high as 1.5 mg/kg ww for molluscs [36].

Twelve of the thirteen studies (Table 2) analysed Pb, with levels ranging from 0.06 ± 0.05 mg/kg dw in Apostichopus japonicus from South Korea [75] to 21.39 ± 2.52 mg/kg dw in Holothuria leucospilota from Qeshm Island in the Persian Gulf [88]. Wen and Hu found trace elements in items at the Chinese market to be within the limits and thus acceptable for human consumption [55]. The concentration of metals in dried products are about 5–10 times higher than in the fresh or rehydrated products consumed. The lack of rigid experimental data on wet weight, or alternatively access to water or moisture contents in the samples or products challenges assessment of the concentration versus health impact as regulative limits. But the Holothuria leucospilota from Qeshm Island in the Persian Gulf [88] and the Eupentacta fraudrix from Peter the Great Bay in the Japan Sea [89], with contents of Pb exceeding the ML, 1.5 mg/kg ww for bivalve molluscs [36], by more than 10 times, are regarded as not suitable for human consumption.

#### 4.2.5. Removal of Heavy Metals from Body Wall of Sea Cucumbers

Lin et al. 2018 [34] studied the bioaccumulation of heavy metals and developed a novel process to remove heavy metals from the body wall of sea cucumbers. They confirmed that bioaccumulation of Hg in sea cucumber is low [90] and that As tended to accumulate in *Apostichopus leucoprocta*. When compared with national limits of food legislation for heavy metals in China [37] (MLs for Pb and As are 0.5 mg/kg, (Aquatic animals and products thereof), the levels of As and Pb exceeded the limits. To remove As and Pb the cucumbers were initially treated with papain and pepsin (0.15% (1:1, *w*/*w*) at 37 °C for 45 min) to hydrolyse the mucus protein of the body wall, followed by soaking in citric acid (0.1 M for 54 h to remove the heavy metals). The resulting product of *Apostichopus leucoprocta* appeared darker, blackish and the structure of the body wall was looser. After soaking for 54 h 98.22 ± 0.91% of As and 94.11 ± 1.08% of Pb were removed and were below 0.5 mg/kg in all samples [34].

### 4.3. Persistent Organic Pollutants (POPs)

Persistent organic pollutants (POPs) include chlorinated (and brominated) aromatic families, such as polychlorinated biphenyls (PCBs), polychlorinated dibenzo-p-dioxins and-furans (PCDDFs), polybrominated diphenyl ethers (PBDEs) and organochlorine pesticides (DDT, toxaphene, chlordane). Some are byproducts of combustion or industrial synthesis (PCDD/Fs) but many have been designed and produced for industrial uses (PCBs, PBDEs) or as agrochemicals (DDT, Lindane, chlordane). The presence of POPs, dioxins and PCBs, both dioxin-like (dl) and non-dioxin-like (ndl) PCBs [91,92], per- and polyfluoroalkyl substances (PFASs) and polycyclic aromatic hydrocarbons (PAHs) in seafood has attracted attention. These are toxic chemicals that accumulate in the food chain from their presence in the environment [93]. Their presence in food and feed has, in general, declined over the last 30 years due to legislative measures and strategies for reduction from public authorities and industry [94,95]. However, due to their long half-lives and persistence, the abundance in the environment is still sustained, and seafood is a major dietary source of these and regulatory levels of content, as well as consumption, apply. Several of these are known to accumulate, as well as biomagnify, in marine organisms. In nature the two processes occur simultaneously, but when assessing cultured species, the accumulation from feed ingredients is the main source of concern.

From the initial screening of abstracts, only ten papers were related to analyses of POPs in sea cucumbers and were thus included for further work. The major proportion were studies of environmental occurrence and bioaccumulation and related mechanisms in sea cucumber, often in areas and depths not used for harvest (Antarctica, 2000 m etc.).

#### 4.3.1. Dioxins and PCBs

Dioxins and PCBs are natural, as well as anthropogenic toxic chemicals that accumulate in the food chain. Among 210 dioxin compounds, 17 are recognized as food hazards. As are twelve of 209 PCBs, the dl-PCBs due to their similar toxicological properties and mechanism, binding to the aryl hydrocarbon receptor (AhR). The European maximum limit of dioxins and dl-PCBs is set at 6.5 pg TE/g ww in muscle of fish and fishery products [70]. To regulate the intake of contaminants in foods, the European Food Safety Authority (EFSA) has defined TWIs. In 2018, the TWI of dioxins and dl-PCBs was lowered from 14 to 2 picogram toxic equivalents (TEQ) per kilogram of body weight per week [96] due to new knowledge regarding adverse effects on semen quality and thus possible effects on male fertility.

None of the papers analysed dioxins, seven papers were related to PCBs (and of these three PAHs) and only a few analysed dl-PCBs. None of these were related to sea cucumber as food, or food products. It would thus be challenging, if not an impossible task to calculate toxic equivalents (TEQ) and compare to limits for content in seafood products. Thus, and surprisingly enough, the possibility to assess the food safety in the context of POPs is limited and future studies are highly warranted.

Due to their lipophilic nature, POPs are usually found dissolved in fatty tissue. Thus, analysis of the levels of lipophilic toxic compounds in EU-regulated foods starts with lipid extraction, in order to isolate lipids from interfering compounds such as water, proteins and carbohydrates [97].

One reason/explanation for this low attention to lipid soluble contaminants, may be the low lipid content (0.09–2.43% ww) of sea cucumbers, with arachidonic acid (AA) and EPA as the major components in many species [23,46]. Thus, compared to other dietary seafood items, sea cucumbers are lean, and not regarded as an optimal dietary source of marine LC-PUFAs. Thus, the lipids as a whole have been less studied.

#### 4.3.2. Polycyclic Aromatic Hydrocarbons

Marine polycyclic aromatic hydrocarbons (PAHs) stem from pyrolytic combustion of organic matter or petroleum-derived sources. PAHs are found in the marine environment, including seawater and sediments, as a result of atmospheric depositions or inputs via rivers. Marine sediments may contain PAHs in high concentration and may pose a risk through bio-accumulation. The metabolic capacity appears to be least developed in filter feeding organisms when compared to other edible species of fish and crustaceans, and thus they may absorb and accumulate PAHs with low elimination rates [98]. In PAHs two or more aromatic rings are linear or clustered in such a manner that makes them hydrophobic. The hydrophobicity increases with size and molecular weight. Low molecular weight PAHs are volatile and degrade easily, while high molecular weight PAHs are persistent and toxic. Specifically, PAH metabolites can bind to DNA and make adducts (benzo[a]pyrene) and cause heritable mutations by disturbing gene expression. The absorption of PAHs is facilitated by their hydrophobic nature. Fatty seafood or food high in lipids, in general, particularly when using food preparation techniques such as grilling and smoking may have an increased content of PAH [99]. The current European legislation provides no specified ML on content of polycyclic aromatic hydrocarbons (PAHs) in Echinodermata, but the EC has set ML for BaP and the sum of BaP, benzo(a)anthracene, chrysene, and benzo(b)fluoranthene (PAH4), as markers for carcinogenic PAHs, in comparable marine food items (bivalve molluscs; ML BaP 5 µg/kg and PAH4 30 µg/kg wet weight) [100].

Only one paper, with data on edible parts of the body wall, of sea cucumbers was found. Khazaali et al., 2016 [101] studied polycyclic aromatic hydrocarbons in sea cucumbers, *Holothuria leucospilota* and *Stichopus hermanni*, from the Persian Gulf. The content varied with six different locations and the range of average concentrations of total PAHs in *Holothuria leucospilota* and *Stichopus hermanni* was 12–505 µg/kg dw and 8–389 µg/kg dw, respectively. Benzo[a]pyrene was detected on one of the sites, the most western in *Holothuria leucospilota* 38.5 ± 19.37 µg/kg dw and at two sites for *Stichopus hermanni* 21.3 ± 2.54 µg/kg dw and 3.8 ± 2.19 µg/kg dw It was not possible to extract PAH4 from the study and the levels are given on a dry weight basis, or 5–10 times higher than on wet weight basis. The lack of experimental data on wet weight, or access to moisture contents challenges assessment of the concentration versus health impact as regulatory limits. During the war (1991) the Persian Gulf environment was heavily contaminated. In addition, most of the world’s oil supply has been transported from the area and offshore oil exploitation is extensive. Other sources of PAHs in this area are combustion of coal, wood, vehicle fuel and waste tires. Nevertheless, as data and knowledge of PAHs in sea cucumber products are almost absent, studies as well as monitoring activities are required, in particular when harvested or cultivated in highly populated or industrialized, areas.

#### 4.3.3. Poly- and Perfluorinated Compounds (PFAS)

The poly- and perfluorinated compounds (PFAS) consist of thousands of various compounds, which accumulate in humans and in the environment. They are used in familiar products, such as non-stick cookware, stain-repellant carpets and furniture, water-repellent textiles, and firefighting foams and over the years they have leached into the ocean and accumulate in the food chain [93]. Perfluorooctane sulfonic acid (PFOS) and perfluorooctanoic acid (PFOA) are regulated through the Stockholm Convention on POPs and within the EU through the Registration, Evaluation, Authorisation and Restriction of Chemicals (REACH). A threshold for the sum of selected PFAS compounds (perfluorohexane sulfonic acid (PFOS) and perfluorononanoic acid (PFOA)), a TWI of 4.4 nanograms per kilogram of body weight per week (ng/kg bw/w) was lately set by EFSA to protect human health [102].

From the initial screening of abstracts, only one, paper was related to analyses of PFAS in sea cucumbers and thus selected for further work [103]. This is a study of bioaccumulation potential, and related mechanisms of the sea cucumber, *Holothura tublosa*. The uptake of PFAS was very rapid, maximum between 22 and 38 days, and the bioaccumulation potential was assessed as high and increased with perfluoroalkyl chain length. Higher concentrations were found in intestines than in gonads, indicating difference in organ distribution, but the body wall—the most relevant material for food—was not analysed. Thus again, it is a challenging task to assess the contribution to intake related to the TWI or food safety, and new knowledge is much required.

#### 4.3.4. Lindane

Lindane, an organochloride chemical, is the commercial name of gamma-hexachlorocyclohexane. Lindane has a variety of applications as an insecticide and has been used as a treatment for lice and scabies. The use of lindane has been banned worldwide and the MRL is 100 ng/g in fish set by the Codex Alimentarius Committee on Pesticide Residues (CCPR). Currently no EU MRLs are set for fish [104]. Shokrzadeh et al., 2009 [105] analysed lindane concentration in cultivated fish as well as the most consumed fish in the Caspian Sea and found levels did not to pose any risk and that the lindane concentration was significantly higher in fish than in the sea cucumbers. This is in accordance with evaluation by EFSA [104]. It is important to monitor lindane in marine animals but for now seafood consumption is not suggested to pose any risks.

### 4.4. Microbiological Hazards

Sea cucumbers may be contaminated with pathogenic microorganisms from their growth environment, as wild or cultivated species, as well as during harvest, processing or handling in general. Hazards generally associated with fishery products (Tae-Yoon et al., 2017, Zhao et al., 2021), although surprisingly, given the commercial value of the products have not been thoroughly studied. Only two papers on microbial load related to food safety [106,107] and three papers on microbial contamination [108,109,110] were found in the systematic screening of abstracts. As always, the sanitary conditions during harvest, transport, processing, and consumption are essential to keep microbial contamination (bacteria and viruses) as low as possible. According to Regulation (EC) No 2073/2005, concerning microbiological criteria for foodstuffs, the only criteria is the more general maximum limit for *Listeria monocytogenes* [111]. No regulation on hygiene at sites for farming sea cucumbers, as for molluscs and sea urchin, were found.

So far, we have discussed the consumption of “bêche-de-mer”, dried body wall, gutted and boiled, the most prevalent food product, and as it is highly processed and boiled, it is safer from the microbial safety perspective. The availability of fresh sea cucumber has increased due to aquaculture and consumption patterns are changing. In order to pursue the original flavour, people increasingly prefer to eat raw ready-to-eat sea cucumbers, in sashimi and sushi, which inevitably causes food safety challenges [107]. Consumption of raw seafood may be the origin of foodborne diseases and outbreaks thus prevention and control measures have to be strengthened as cuisines develop. Pathogens connected to foodborne poisoning from raw seafood consumption may include *Bacillus cereus*, *Escherichia coli*, *Listeria monocytogenes*, *Salmonella* spp., *Vibrio parahaemolyticus* and *Vibrio cholerae* [106].

Zhao et al., 2021 found three different isolates of *Bacillus cereus* on sea cucumbers in three coastal aquaculture sites in northeastern China. They demonstrated that all three *B. cereus* isolates possessed multiple potential virulence factors, which indicates the urgent need to improve microbial detection in aquatic products to ensure seafood safety in China. A higher diversity in the microbial load was found at sites in Korea [106] and both authors discuss the urgent need to develop management and monitoring strategies to improve food safety, in particular, with raw sea cucumber consumption.

#### Antibiotics

Of the three papers related to antimicrobials, two were connected to the detection of nitrofuran antibiotic drugs [109,110]. Highly effective and prohibited for years but still widely found in animal food products. A more general paper [108] screening for residues of antibiotics in Chinese waters, found ten antibiotics in sea cucumbers. Sulfonamides are the predominant antibiotics followed by macrolides and fluoroquinolones. Possible food safety risks were assessed by calculation of hazard quotients and the authors concluded that there were no obvious human health risks associated with the consumption of sea cucumbers.

### 4.5. Physical Hazards

Only one of the of the papers from the initial screening was related to physical hazards, the challenges of micro and nano sized plastics in ocean environments [112]. Micro- and nano plastics are known physical hazards in seafood [113]. Mohsen et al. studied microplastic ingestion by farmed sea cucumber. The microplastics, microfibres from the habitat, were detected in the intestines and the coelomic fluid [112]. Results from examining gastrointestinal systems are of limited relevance to food safety of sea cucumbers, since only the body wall is consumed directly by humans. The viscera or all intestines are removed and not consumed without further processing into extracts or feed components [112]. Plastics can serve as vehicles for chemical contaminants that can be harmful to the biota, and contribute to the human exposure to these compounds. Such chemicals may either be inherent to the plastic or absorbed from the environment. Further studies are required to determine if desorption of chemicals may pose a risk to consumers of seafood [79,112,114].

The physical hazards of concern in sea cucumbers will thus mainly come from the production line, process packaging, transport and preparation of meals. As already noted, the product consumed is mainly the body wall, eviscerated, washed and dried and through this process physical hazards from the habitat or the environment and the harvest will largely be removed. Such physical hazards include material from the production process of specific products and packaging material such as metal and glass.

## 5. Conclusions

A high proportion of the selected papers concerned heavy metals and metalloid hazards, such as Hg, Cd, Pb, and As. Unfortunately, no specific maximum limits have been set for contents of sea cucumbers. Thus, the levels present in sea cucumbers were compared with limits for food items from aquatic animals, and if possible and preferable, other sedentary filter feeders such as bivalve molluscs.

With regard to Hg and Cd concentrations, none of the samples exceeded the limits set by the European Commission or National Standard of China, while for Pb a few samples, from highly polluted industrial areas, exceeded the limits and thus were assessed as not suitable for human consumption.

There are no maximum limits for total As in seafood. The content of As varies significantly between sea cucumbers species. The less toxic organic form is generally the most abundant As species in sea cucumbers. One exception being the *Cucumaria frondosa* harvested in Canadian sea cucumber fisheries, where iAs represented around 70% of the total *recovered* As. Making an assessment of health impacton the basis of one study on content of iAs in sea cucumber is challenging. More studies on speciation of As is thus highly warranted.

Sea cucumbers are filter feeders and may take up toxin producing algae present in surrounding environments or is close habitat. Surprisingly enough, given the commercial value of the sea cucumber, none of the retrieved papers had data on biotoxins related to food safety. Only a few of the retrieved papers had data on contaminants such as POPs. None of the retrieved papers analysed dioxins, seven papers were related to PCBs and only a few analysed dl-PCBs. None of these were related to sea cucumber as food, or food products. One paper presented data on content of PAH and BaP, in the edible part of sea cucumber but the assessment was challenged by the lack of data on PAH4 and data given on dry weight while the maximum limits in the regulatory framework are given on a wet weight basis. A study was performed on more emerging contaminants, PFAS in sea cucumbers, but the analysis was not performed on the preferred edible part the body wall.

Consumption of sea cucumbers is expanding and the high exploitation has led to decline in populations of sea cucumbers. Aquaculture, together with fisheries on new species in new regions eases the demand. This change to new regions and to new species may pose a challenge to the food safety of sea cucumber products. The content of contaminants varies both with species and the degree of industrial activity in the region. Research aiming at the impact of industrial activities on contents of POPs and in particular emerging contaminants as PFAS is thus highly warranted.

The availability of fresh sea cucumber has increased due to aquaculture. In order to pursue the original flavour, some consumers are reported to prefer to eat raw sea cucumber products, sashimi and sushi, which inevitably causes challenges from a microbial food safety perspective.

Lack of data on daily intake of sea cucumber (quantities, frequencies, product used) add to the challenge of assessing health risks associated with sea cucumber consumption. Human health risk assessment is thus not available and the data gaps, such as more rigid monitoring and analysis of contaminants, and data of the consumption, needs to be undertaken to enable a valid health risk assessment of sea cucumber consumption.

Regulations are not in place to protect the consumer, facilitating the production of safe seafood. Altogether, this highlights the need for strategies to improve the knowledge, increase available data related to food safety and develop appropriate guidelines and regulations on the production, processing and utilization of sea cucumbers. In addition, there is a requirement for well-designed controlled trials to confirm pharmaceutical and nutraceutical benefits of sea cucumbers or their extracts.

## Figures and Tables

**Table 1:** Proximate composition (%) of fresh and dried sea cucumber species presented in ranges or as mean +/− standard deviation, or as presented in original works.

Species	Water	Ash	Protein	Fat	Ref
Fresh sea cucumber					
*Apostichopus japonicus*	90.7 ± 0.35	3.2 ± 0.4	3.4 ± 0.1	0.1 ± 0.1	Rasyid et al. [23]
*Cucumaria frondosa*	90.5	0.8	5.5	3.5	Hossain et al. [24]
*Holothuria poli*	81.2 ± 0.4	7.9 ± 0.9	8.7 ± 1.2	0.2 ± 0.0	Rasyid et al. [23]
*Holothuria scabra*	84.5–87.2	3.59–11.1	5.8–9.5	0.2–0.8	Özer et al., Rasyid et al. [23,44]
*Holothuria tubulosa*	81.4–86.5	4.1–6.1	8.0–10.2	1.6–1.9	Künili & Çolakoğlu, Rasyid et al. [23,45]
Dried sea cucumber					
*Actinopyga caerulea*	0.81 ± 0.03	28.4 ± 0.3	56.9 ± 0.4	10.1 ± 0.25	Wen et al. [43]
*Actinopyga mauritana*	11.6 ± 0.31	15.4 ± 0.2	63.3 ± 0.4	1.4 ± 0.02	Wen et al. [43]
*Bohadschia argus*	13.0 ± 0.26	17.7 ± 0.2	62.1 ± 0.4	1.1 ± 0.01	Wen et al. [43]
*Holothuria fuscogilva*	11.6 ± 0.28	26.4 ± 0.3	57.8 ± 0.4	0.3 ± 0.01	Wen et al. [43]
*Holothuria fuscopunctata*	7.0 ± 0.14	39.6 ± 0.2	50.1 ± 0.4	0.3 ± 0.01	Wen et al. [43]
*Holothuria polii*	22.0 ± 3.1	48.2 ± 1.1	37.0 ± 0.6	0.6 ± 0.1	Rasyid et al. [23]
*Holothuria scabra*	9.1 ± 0.3	5.8 ± 0.4	72.3 ± 0.6	2.0 ± 0.1	Rasyid et al. [23]
*Holothuria tubulosa*	16.2 ± 1.5	46.4 ± 0.5	44.6 ± 1.0	0.7 ± 0.1	Rasyid et al. [23]
*Stichopus herrmanni*	10.2 ± 0.32	37.9 ± 0.3	47.0 ± 0.4	0.8 ± 0.02	Wen et al. [43]
*Theleonata ananas*	15.1 ± 0.29	25.1 ± 0.3	55.2 ± 0.4	1.9 ± 0.01	Wen et al. [43]
*Theleonata anax*	1.2 ± 0.06	39.2 ± 0.3	40.7 ± 0.3	9.9 ± 0.27	Wen et al. [43]

## Data Availability

Data is contained within the article.
